# Clinical implications of the plasma EphA2 receptor level in critically ill patients with septic shock

**DOI:** 10.1038/s41598-017-17909-7

**Published:** 2017-12-14

**Authors:** Su Hwan Lee, Ju Hye Shin, Joo Han Song, Ah Young Leem, Moo Suk Park, Young Sam Kim, Joon Chang, Kyung Soo Chung

**Affiliations:** 1 Division of Pulmonary and Critical Care Medicine, Department of Internal Medicine, Ewha Medical Research Institute, Ewha Womans University School of Medicine, Seoul, Republic of Korea; 2 Division of Pulmonology, Department of Internal Medicine, Severance Hospital, Institute of Chest Diseases, Yonsei University College of Medicine, Seoul, Korea; 3 Yonsei University College of Medicine, Seoul, Republic of Korea

## Abstract

The Eph/ephrin receptor ligand system is known to play a role in inflammation induced by infection, injury, and inflammatory diseases. The present study aimed to evaluate plasma EphA2 receptor levels in critically ill patients with sepsis. This study was a prospective cohort study evaluating samples and clinical data from the medical intensive care unit (MICU) of a 2000-bed university tertiary referral hospital in South Korea. Positive correlations of the plasma EphA2 receptor level with the acute physiology and chronic health evaluation (APACHE) II score and the sequential organ failure assessment (SOFA) score were observed. The area under the curve (AUC) for the plasma EphA2 receptor level on a receiver operating characteristic curve was 0.690 (95% confidence interval [CI], 0.608–0.764); the AUCs for the APACHE II score and SOFA scores were 0.659 (95% CI, 0.576–0.736) and 0.745 (95% CI, 0.666–0.814), respectively. A Cox proportional hazard model identified an association between an increased plasma EphA2 receptor level (>51.5 pg mL^−1^) and increased risk of 28-day mortality in the MICU (hazard ratio = 3.22, 95% CI, 1.709–6.049). An increased plasma EphA2 receptor level was associated with sepsis severity and 28-day mortality among sepsis patients.

## Introduction

The progression of infection-induced sepsis leads to organ dysfunction and is associated with a high rate of mortality^1^. Although the sepsis mortality rate has significantly decreased over the years, this condition remains difficult to treat^2^. Furthermore, the reported incidence of sepsis has increased because of several factors, including an increase in elderly populations with more comorbidities^3–6^. In addition, half of all sepsis patients are managed in intensive care units (ICUs), and more than 25% of cases end in death^7,8^.

Vascular endothelial dysfunction plays a key role in the organ failure and mortality associated with sepsis^2^. Sepsis alters the endothelial barrier function, thus enhancing the passage of water, soluble proteins, and cellular components from the blood to the tissues^9^. In addition to conventional treatments such as early antibiotics and conservative management, the use of several anti-inflammatory or immunomodulatory therapies has been studied to combat the pro-inflammatory stage of sepsis^10^. However, several attempts to target tumor necrosis factor (TNF)-α, interleukin (IL)-1 β, toll-like receptors, and endotoxin have not been effective in clinical trials^10–12^.

Eph receptors comprise the largest family of tyrosine kinase receptors and, with ephrin ligands, are involved in cell-to-cell communication. Interactions between Eph receptors and ephrin ligands affect the pathogeneses of many conditions including wound healing, ischemia reperfusion injury, nerve injury, endothelial injury, and epithelial injury^13–16^. Eph receptors and ephrin ligands also regulate important processes during embryonic neuronal development, angiogenesis, and oncogenesis^13,16^. Recently, many studies have focused on the complex roles of Eph and ephrin in malignancy^17,18^. According to several studies, the EphA2 receptor and ephrinA1 ligand also affect inflammation via vascular endothelial injury. In an *in vivo* study in rats, ephrin A1 induced histological endothelial disruption in the lung and blocking EphA2 signaling markedly reduced leakage of albumin^13,16,19^. Another study showed that EphA2 activation induced expression of vascular cell adhesion molecule-1 and E-selectin^20^. The EphA2 receptor may be associated with sepsis due to the important role of endothelial injury in sepsis^21,22^. However, clinical studies investigating the association between the EphA2 receptor and sepsis are currently lacking.

We have found that the plasma EphA2 receptor level is elevated in sepsis patients, and therefore, we investigated the clinical implications of the plasma EphA2 receptor level with regard to sepsis. The primary outcome of the study was the association between the plasma EphA2 receptor level and all-cause 28-day mortality among sepsis patients. The secondary outcomes were an evaluation of the correlations between plasma EphA2 receptor levels and severity of sepsis as determined by the acute physiology and chronic health evaluation (APACE) II score and the sequential organ failure assessment (SOFA) score^23,24^. Because EphA2 receptor blocking antibodies are already undergoing clinical trials for cancer^25–28^, we also investigated the plasma EphA2 receptor level as a potential marker for therapeutic monitoring in sepsis patients.

## Results

### Demographic characteristics of the overall study population

**Figure 1:**
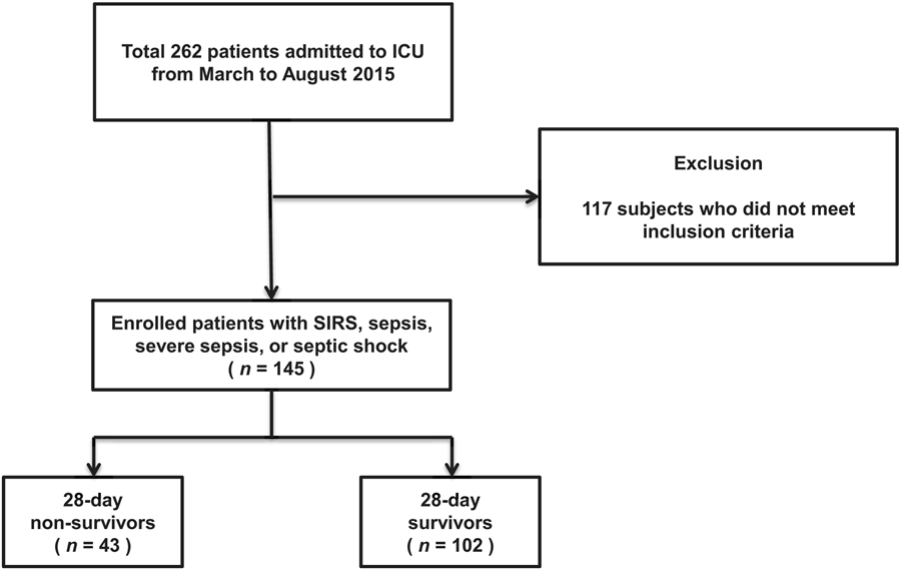
Flow diagram of the study population.

A total of 262 patients were admitted to our medical ICU (MICU) between March 2015 and August 2015. Of these, 145 patients presented with systemic inflammatory response syndrome (SIRS), sepsis, severe sepsis, or septic shock and agreed to participate in this study (Fig. 1). The baseline characteristics of the study population are represented in Table 1. The patient population mostly consisted of older individuals (median age, 70 years) with a higher proportion of men than women (65.5% *vs*. 35.5%). Patients with severe sepsis and septic shock accounted for 73.1% of the study population. The median score on the Charlson Comorbidity Index was 3 (interquartile range [IQR], 2–5), and only 6 patients did not present with comorbidities. The incidence of acute respiratory distress syndrome (ARDS) and positive blood culture were 18.6% and 25.5%, respectively. The most common site of infection was the lung (59.3%), followed by the gastrointestinal tract (10.3%). The median acute physiology and chronic health evaluation (APACHE) II score was 25 (IQR, 18–31), and the median sequential organ failure assessment (SOFA) score was 10 (IQR, 7–13). Overall, 43 patients (29.7%) died within 28 days of admission to the MICU.

**Table 1:** The characteristics of the patients.

**Variables**	**Total**
	**(** ***n*** = **145)**
Age (years)	70 (61–77)
Sex (male)	95 (65.5)
APACHE II score	25 (18–31)
SOFA score	10 (7–13)
ARDS	27 (18.6)
Severe sepsis and septic shock	106 (73.1)
Positive of blood culture	37 (25.5)
Charlson Comorbidity Index	3 (2–5)
CHF	24 (16.6)
DM	45 (31.0)
CKD or ESRD	33 (22.8)
CRP (mg L^−1^)	87.3 (22.3–141.2)
Procalcitonin (ng mL^−1^)	0.8 (0.2–6.2)

Values are expressed as *n* (%) or median (interquartile range) unless otherwise indicated; APACHE, acute physiology and chronic health evaluation; SOFA, sequential organ failure assessment; ARDS, acute respiratory distress syndrome; CHF, congestive heart failure; DM, diabetes mellitus; CKD, chronic kidney disease; ESRD, end stage renal disease; CRP, C-reactive protein.

### Comparison of characteristics between 28-day survivors and 28-day non-survivors

This study divided patients into two groups according to 28-day mortality: patients in the case group died within 28 days of admission to the MICU, whereas those in the control group survived longer than 28 days. The results of this inter-group comparison are shown in Table 2. The groups did not differ significantly with regard to sex, median age, and median body mass index (BMI). Regarding clinical parameters, the groups did not differ in terms of the rate of positive blood cultures and median scores on the Charlson Comorbidity Index. However, the non-survivor group had higher rates of ARDS (32.6% *vs*. 12.7% for survivors, *P* = 0.005) and a sepsis status above severe (86.0% *vs*. 67.6% for survivors, *P* = 0.022). The median APACHE II score (29 *vs*. 24, *P* = 0.002) and median SOFA score (13 *vs*. 8, *P* < 0.001) were also significantly higher in the non-survivor group relative to the survivor group. Regarding laboratory parameters, no differences were observed between survivors and non-survivors in the median levels for C-reactive protein (CRP) (73.5 mg mL^−1^ vs. 97 mg mL^−1^, *P* = 0.347) and procalcitonin (0.5 ng mL^−1^ vs. 1.8 ng mL^−1^, *P* = 0.181) on the day of admission. However, the median plasma EphA2 receptor level was significantly higher in the non-survivor group than in the survivor group (78.5 pg mL^−1^ vs. 33.3 pg mL^−1^, *P* < 0.001).

**Table 2:** Comparison of Characteristics Between 28-day Survivors and 28-day Non-survivors (n = 145).

Variables	Survivor	Non-survivor	*p*
	(*n* = 102)	(*n* = 43)	
Sex (male)	67 (65.7)	28 (65.1)	0.947
Age (year)	69 (57.8–77.0)	72 (63.1–84.2)	0.202
Body mass index (kg m^−2^)	20.6 (18.9–26.6)	21.7 (19.4–30.0)	0.081
Clinical parameters			
ARDS	13 (12.7)	14 (32.6)	0.005
Severe sepsis or septic shock	69 (67.6)	37 (86.0)	0.022
Positive blood culture	24 (23.5)	13 (30.2)	0.398
Charlson Comorbidity Index	3 (1.0–4.3)	3 (2.0–5.0)	0.080
APACHE II score	24.0 (16.8–29.2)	29.0 (20.0–36.0)	0.002
SOFA score	8.0 (6.0–11.3)	13.0 (10.0–16.0)	<0.001
Laboratory parameters			
CRP (mg mL^−1^)	73.5 (17.5–158.4)	97.0 (47.0–186.0)	0.347
Procalcitonin (ng mL^−1^)	0.5 (0.1–6.3)	1.8 (0.5–5.7)	0.181
DNI (%)	3.2 (1.1–6.9)	3.8 (2.1–13.3)	0.202
RDW (%)	15.0 (14.1–16.5)	17.1 (15.9–19.6)	<0.001
EphA2 (pg mL^−1^)	33.3 (18.8–71.0)	78.5 (36.3–164.5)	<0.001
IL-1β (pg mL^−1^)	14.5 (12.0–26.8)	19.0 (12.0–31.3)	0.327
IL-10 (pg mL^−1^)	84.8 (27.5–127.9)	99.5 (28.5–465.5)	0.113
IL-18 (pg mL^−1^)	408.6 (267.9–751.3)	835.5 (467.8–1719.0)	<0.001
IL-6 (pg mL^−1^)	311.6 (109.3–1123.6)	2329.2 (247.5–7570.0)	0.002
TNF-α (pg mL^−1^)	41.0 (27.7–70.2)	45.0 (27.3–100.3)	0.320
IP-10 (pg mL^−1^)	618.6 (217.2–2205.9)	1597.0 (499.3–5468.3)	0.013

Values are expressed as *n* (%) or median (interquartile range) unless otherwise indicated; ARDS, acute respiratory distress syndrome; APACHE, acute physiology and chronic health evaluation; SOFA, sequential organ failure assessment; DNI, delta neutrophil index; RDW, red cell distribution width; IL, interleukin; TNF, tumor necrosis factor; IP, induced protein.

### Association between the plasma EphA2 receptor level and severity or mortality

**Figure 2:**
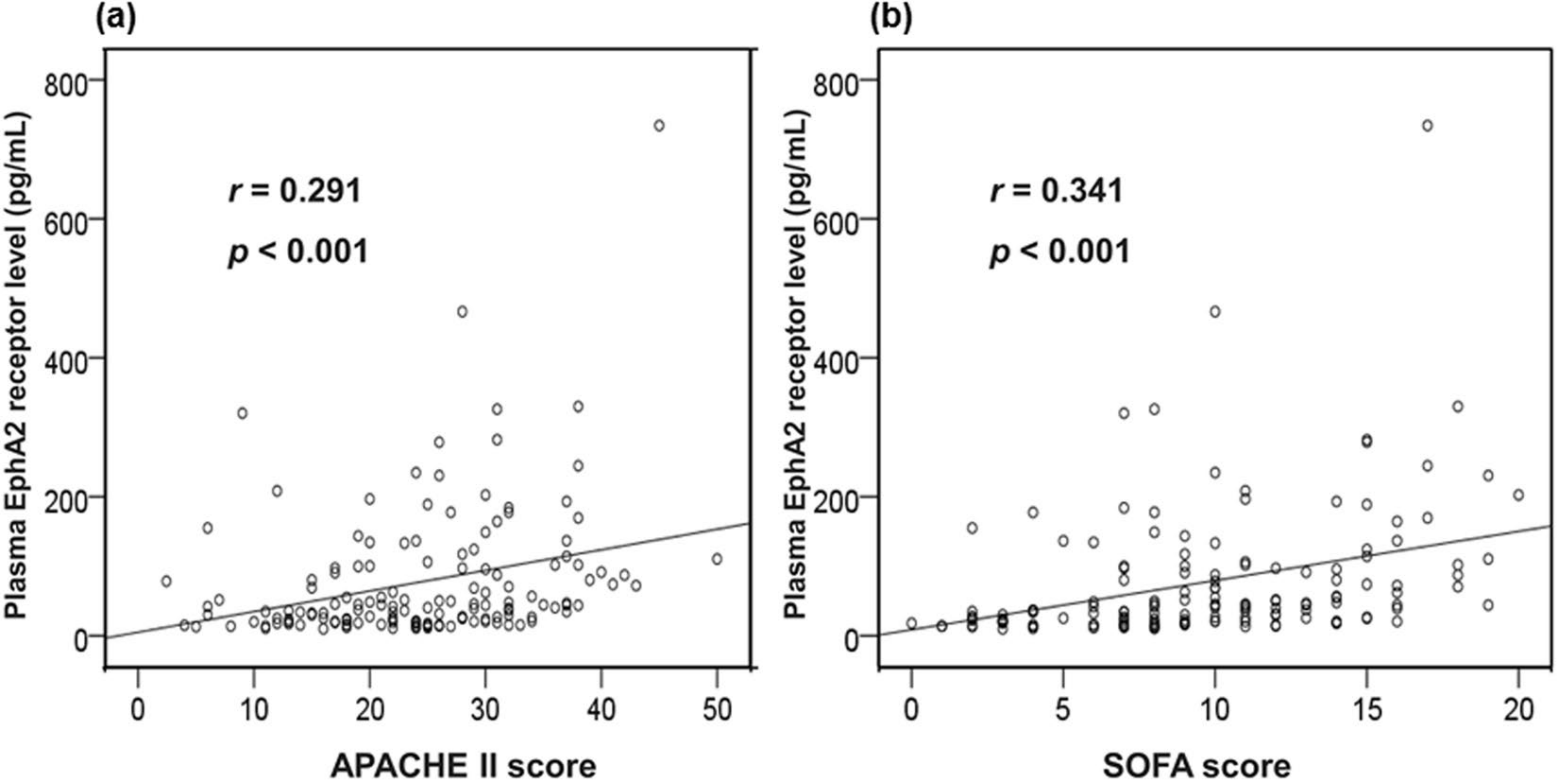
Positive correlations were observed between the plasma EphA2 receptor level and the (**a**) APACHE II and (**b**) SOFA scores.

**Figure 3:**
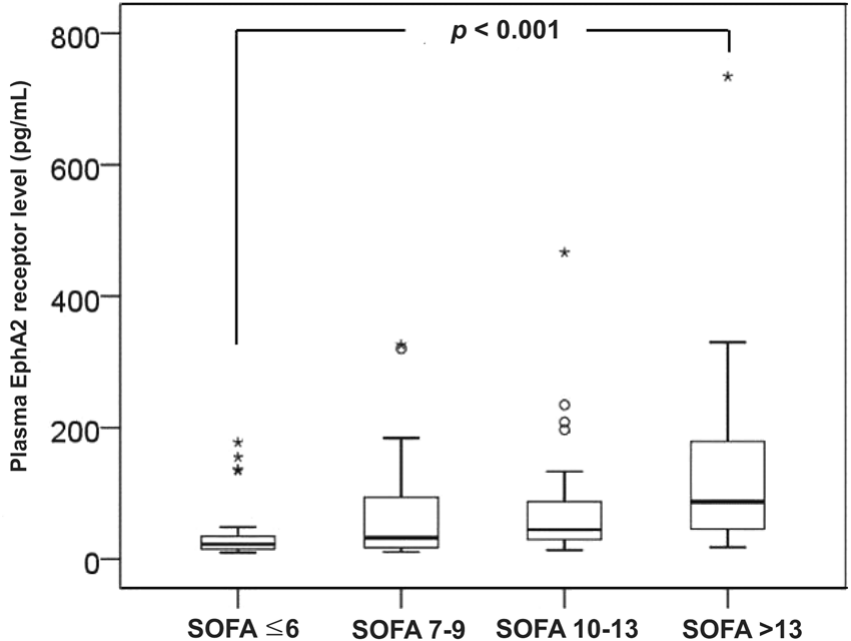
Stratification of patients according to the interquartile ranges of SOFA scores revealed an increasing trend in SOFA scores with increasing plasma EphA2 receptor levels.

**Figure 4:**
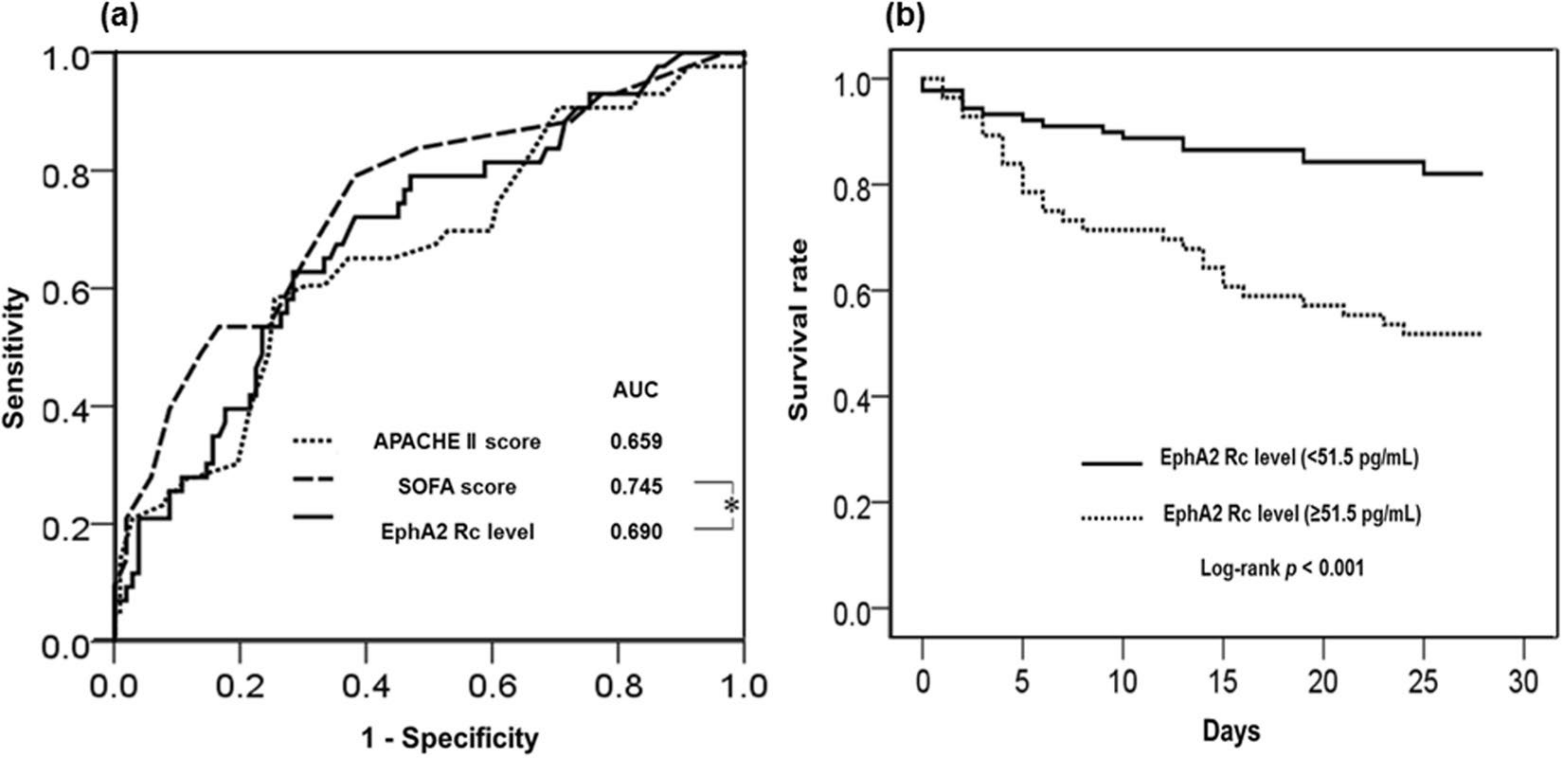
(**a**) In ROC curve comparisons, the AUC of the plasma EphA2 receptor level and SOFA scores (0.690 *vs*. 0.745, *P* = 0.256) and APACHE II scores (0.690 *vs*. 0.659, *P* = 0.605) did not differ significantly. (**b**) Kaplan-Meier survival analysis showed that the 28-day mortality of patients with plasma EphA2 receptor level ≥51.5 pg mL^−1^ was higher than that of patients with plasma EphA2 receptor level <51.5 pg mL^−1^.

Positive correlations were observed between the plasma EphA2 receptor level and the APACHE II (*r* = 0.291, *P* < 0.001) and SOFA (*r* = 0.341, *P* < 0.001 Fig. 2) scores. Stratification of patients according to the IQRs for SOFA scores revealed an increasing trend in SOFA scores with increasing plasma EphA2 receptor levels (SOFA score <6 *vs*. SOFA score >13, *P* < 0.001; Fig. 3). The Area under the curve (AUC) for the plasma EphA2 receptor level on a receiver operating characteristic (ROC) curve was 0.690 (95% confidence interval [CI], 0.608–0.764); the AUCs for the APACHE II score and SOFA score were 0.659 (95% CI, 0.576–0.736) and 0.745 (95% CI, 0.666–0.814), respectively. In ROC curve comparisons, the AUC of the plasma EphA2 receptor level and SOFA score (0.690 *vs*. 0.745, *P* = 0.256) and APACHE II score (0.690 *vs*. 0.659, *P* = 0.605) did not differ significantly. However, the AUC of the SOFA score and APACHE II score differed significantly (0.745 *vs*. 0.659, *P* = 0.034; Fig. 4a).

A cut-off value of >51.5 pg mL^−1^ for the plasma EphA2 receptor level was determined to predict 28-day mortality on the ROC (sensitivity, 62.8%; specificity, 71.6%). Patients were divided into two groups according to this plasma EphA2 receptor cut-off level of 51.5 pg mL^−1^ and subjected to a Cox proportional hazard model analysis involving several demographic and clinical characteristics and substituted plasma EphA2 receptor levels (Table 3). In the univariate analysis, an increased plasma EphA2 receptor level (>51.5 pg mL^−1^; hazard ratio, 3.41; 95% CI, 1.750–6.636) was associated with 28-day mortality. In the multivariate analysis, sex, age, BMI, positive blood culture and sepsis status higher than severe were not found to be associated with 28-day mortality; in contrast, ARDS (hazard ratio, 2.02; 95% CI, 1.051–3.389) and an increased plasma EphA2 receptor level (>51.5 pg mL^−1^; hazard ratio, 3.22; 95% CI, 1.709–6.049) were associated with increased 28-day mortality in the MICU. Figure 4b shows the survival rates of the groups classified according to plasma EphA2 receptor levels; these survival rates differed significantly (*P* < 0.001).

**Table 3:** Cox proportional hazard model of plasma EphA2 level and 28-day mortality.

Variable	Univariate analysis			Multivariate analysis		
	HR	95% CI	*p*	HR	95% CI	*p*
Sex (male)	1.22	0.641–2.316	0.546			
Age (year)	1.02	0.998–1.044	0.076	1.02	0.999–1.045	0.056
BMI (kg m^−2^)	1.04	0.960–1.131	0.320			
ARDS	1.96	0.996–3.838	0.051	2.02	1.051–3.887	0.035
Positive Blood culture	0.68	0.334–1.375	0.281			
EphA2 (≥51.5 pg mL^−1^)	3.41	1.750–6.636	<0.001	3.22	1.709–6.049	<0.001
Severe sepsis and septic shock	2.48	0.992–6.205	0.052	2.32	0.970–5.541	0.059

HR, hazard ratio; CI, confidence interval; BMI, body mass index; ARDS, acute respiratory distress syndrome.

## Discussion

In the current study, we investigated potential associations of the plasma EphA2 receptor level with sepsis severity and 28 day-mortality among sepsis patients in the MICU. The main finding of this study was that the plasma EphA2 receptor level correlates with sepsis severity scores and increased mortality among sepsis patients.

The APACHE II score and SOFA score are commonly used for initial severity assessments in sepsis patients^23,29^. The plasma EphA2 receptor level was comparable to these scores. The results of this analysis consistently indicated that an increased plasma EphA2 receptor level was related to the initial severity of sepsis. A higher SOFA score is associated with increased patient mortality^24^. In this study, plasma EphA2 receptor levels were evaluated in groups according to interquartile ranges of SOFA scores (Fig. 3). The result showed that a higher plasma EphA2 receptor level was associated with mortality in sepsis patients. The AUC of the ROC curve and results of the Cox hazard proportional model supported this association. Furthermore, we used a plasma EphA2 receptor cut-off value to discriminate mortality risk according to the Cox hazard proportional model. As a result, a plasma EphA2 receptor level >51.5 pg mL^−1^ was associated with a significant increase in mortality (hazard ratio, 3.22).

As mentioned previously, many studies have focused on the complex roles of the Eph receptor and ephrin ligand in malignancy^30,31^. Several studies have reported associations of EphA2 with multiple oncogenic signaling pathways such as MAP/ERK, phosphoinositide 3-kinase, E-cadherin, and integrin/FAK/paxillin^30,32–34^. In the field of oncology, the EphA2 receptor has been studied as a therapeutic target and is being investigated in an ongoing clinical trial in patients with advanced malignancies^25–28^. Tumor development, progression, and therapeutic responses may be affected by inflammation^17^. Cancer and infectious disease have many similarities. For example, inhibitors of programmed cell death ligand 1, which were developed for patients with cancer, have also been actively investigated as therapeutic agents for chronic infection and/or sepsis^18^. Accordingly, it is noteworthy that the Eph2 receptor and ephrin ligand also affect inflammation through vascular endothelial injury^13,16,19^. EphA2 and other inflammatory mediators, such as TNF-α and interferon (IFN)-γ, upregulate NF-κB, thus increasing intercellular adhesion molecule-1 expression and facilitating leukocyte migration and attachment^19,35^. Several emerging studies have shown a link between the EphA2 receptor and inflammation^36–40^. Animal models of lipopolysaccharide (LPS)-induced pneumonia have demonstrated an increase in the EphA2 receptor level after LPS exposure^37^. Another study reported the increased expression of EphA2 receptor with lung injury and found that EphA2 receptor blockade reduced lung injury and the passage of fluids and inflammatory cells^41^. EphA2 receptor signaling is associated with Src family kinases, mitogen activated protein kinase, p-21 activated kinase, post-synaptic density protein 95-dependent pathways, chemokine pathways, heterotrimeric G-protein pathways, and integrin-mediated pathways^19,22^. A recent study reported that the p-21 activated kinase was strongly associated with endothelial barrier in a sepsis murine model^22^. Given the results of this study and others, EphA2 receptor blockade may be a reasonable treatment target for reducing endothelial injury in sepsis patients. Unlike malignancy, however, sepsis does not involve a target lesion. Therefore, an appropriate blood marker with which to assess the therapeutic drug effect is needed if EphA2 receptor blocking is to be used as a therapeutic target. The plasma EphA2 receptor level may be useful to evaluate the effect of treatment in this setting.

The strengths of this study include the confirmed measurement of blood plasma EphA2 receptor levels and the identification of an association between human plasma EphA2 receptor levels and sepsis. These results provide clinical evidence for the use of EphA2 receptor as a therapeutic target in sepsis and provide a potential tool for monitoring responses to EphA2 receptor blocker treatment in sepsis patients.

However, our study has several limitations. First, this was a small study of plasma EphA2 receptor levels in sepsis patients at a single center without pediatric patients. However, this study is the first clinical study to use human blood samples to evaluate the relationship between plasma EphA2 receptor levels and sepsis. Previously, other studies investigated the relationship between the EphA2 receptor and inflammation. Most of these studies used *in vitro* or animal models^36,37,39,42^. However, no clinical proof of sepsis was provided. Second, the plasma EphA2 receptor level measurements have not been validated. The importance of proteases in regulating the Eph receptor and the ephrin ligand family has been shown^43^; it is thought that the proteases that activate the Eph receptor and the ephrin ligand family also degrade the Eph A2 receptor into the cleaved form, which in turn increases EphA2 receptors in the plasma. However, it is unclear whether the measured EphA2 receptor reflects the soluble or membrane-bound form due to limitation of the kit used in this study. Furthermore, *in vitro* and *in vivo* evidence that clearly supports an association between plasma EphA2 receptor signaling and sepsis is rare. Third, we did not analyze serial EphA2 receptor levels because we were unable to collect serial blood samples from the patients. Therefore, additional studies investigating the measurement of plasma EphA2 receptor levels in sepsis patients and evidence of an association between EphA2 receptor signaling and sepsis are needed.

## Conclusion

An increased plasma EphA2 receptor level was associated with sepsis severity and 28-day mortality among sepsis patients. The plasma EphA2 receptor may be considered a potential therapeutic target in sepsis patients. The EphA2 receptor level may be used for treatment monitoring in sepsis patients treated with an EphA2 receptor blocker.

## Methods

### Study design, patients, and clinical setting

This was a prospective cohort study of patients and collected samples from the MICU of Severance Hospital, a 2000-bed (30-bed MICU) university tertiary referral hospital in Seoul, South Korea. The study considered 262 consecutive patients aged >19 years who were admitted to the MICU between March 2015 and August 2015. The study ultimately enrolled a total of 145 patients who presented with SIRS, sepsis, severe sepsis, or septic shock as defined by the American College of Chest Physicians/Society of Critical Care Medicine Consensus Conference^5,44^. All patients presenting with SIRS, sepsis, severe sepsis, or septic shock were treated according to the guidelines of the Surviving Sepsis Campaign^5^.

### Clinical data and blood sample collection

Data of all patients admitted to the MICU were collected from the hospital electronic medical records and their blood samples. The severity of each patient’s condition was classified according to two different scoring systems: The SOFA and APACHE II scores were calculated^23,29^. In addition, the Charlson Comorbidity Index was used to evaluate patients’ comorbidities^45^. Clinical parameters such as the development of ARDS, blood culture positivity, 28-day mortality, and other demographic characteristics were evaluated.

Venous blood samples were collected within 24 hours after admission to the MICU though central lines into tubes containing ethylenediaminetetraacetic acid. Simultaneously, we determined the CRP, procalcitonin, creatinine, and albumin levels, blood culture results, white blood cell and platelet counts, and APACHE II and SOFA scores. Plasma was prepared by centrifugation for 15 minutes at 800 × *g* and 4 °C. Supernatants from centrifuged blood were immediately aliquoted and stored at −80 °C until the analysis was performed. Plasma levels of the EphA2 receptor and other cytokines (IL-1β, IL-10, IL-18, IL-6, TNF-α, and interferon gamma induced protein-10) were measured using the Human Magnetic Luminex^®^ Screening Assay Kit (R&D Systems, Inc., Minneapolis, MN, USA). The kit detects all forms of the EphA2 receptor including the soluble, membrane-bound, phosphorylated and non-phosphorylated forms All samples and standards were assayed in duplicate using the Luminex 200^TM^ System (Merck Millipore, Darmstadt, Germany).

### Ethical approval

The study protocol was submitted as an ICU cohort and approved by the Institutional Review Board (IRB) of Severance Hospital (IRB number: 4-2013-0585). All study procedures were performed in accordance with the relevant guidelines and regulations. In addition, this study was performed in compliance with the principles set forth in the Declaration of Helsinki.

### Statistical analysis

Data are described as medians with IQRs. The chi-squared and Fisher’s exact tests or the Mann–Whitney U test was used to assess differences between the two groups. The Kruskal–Wallis test was used to compare three or more groups of a qualitative parameter. Pearson correlation analyses were performed to estimate associations between the biomarkers and the APACHE II and SOFA scores. AUC analyses of ROC curves were performed to compare the plasma EphA2 receptor level, APACHE II score, and SOFA score. Subsequently, the 28-day survivor and non-survivor groups were analyzed using a Cox proportional hazard model with a plasma EphA2 receptor level cut-off value to predict 28-day mortality according to the ROC and several variables. The Kaplan–Meier method was used to report survival curves that were analyzed using the log-rank test. In all cases, a p-value of <0.05 was considered statistically significant. SPSS version 20 (IBM, Armonk, NY, USA) was used for statistical analyses; AUC analyses of ROC curves were performed using MedCalc software (version 16.4.3; MedCalc, Oostende, Belgium).
